# Defining Acute Coronary Syndrome through Metabolomics

**DOI:** 10.3390/metabo11100685

**Published:** 2021-10-06

**Authors:** Arun Surendran, Negar Atefi, Hannah Zhang, Michel Aliani, Amir Ravandi

**Affiliations:** 1 Cardiovascular Lipidomics Laboratory, St. Boniface Hospital, Albrechtsen Research Centre, Winnipeg, MB R2H 2A6, Canada; surendas@myumanitoba.ca (A.S.); atefin@myumanitoba.ca (N.A.); zhangh22@myumanitoba.ca (H.Z.); 2 Mass Spectrometry and Proteomics Core Facility, Rajiv Gandhi Centre for Biotechnology, Thiruvananthapuram 695014, Kerala, India; 3 Department of Physiology and Pathophysiology, Rady Faculty of Health Sciences, University of Manitoba, Winnipeg, MB R2H 2A6, Canada; 4 Faculty of Agricultural and Food Sciences, Rady Faculty of Health Sciences, University of Manitoba, Winnipeg, MB R2H 2A6, Canada; Michel.Aliani@umanitoba.ca; 5 Section of Cardiology, Department of Medicine, Rady Faculty of Health Sciences, University of Manitoba, Winnipeg, MB R2H 2A6, Canada

**Keywords:** metabolomics, lipidomics, acute coronary syndrome, ischemia/reperfusion injury

## Abstract

As an emerging platform technology, metabolomics offers new insights into the pathomechanisms associated with complex disease conditions, including cardiovascular diseases. It also facilitates assessing the risk of developing the disease before its clinical manifestation. For this reason, metabolomics is of growing interest for understanding the pathogenesis of acute coronary syndromes (ACS), finding new biomarkers of ACS, and its associated risk management. Metabolomics-based studies in ACS have already demonstrated immense potential for biomarker discovery and mechanistic insights by identifying metabolomic signatures (e.g., branched-chain amino acids, acylcarnitines, lysophosphatidylcholines) associated with disease progression. Herein, we discuss the various metabolomics approaches and the challenges involved in metabolic profiling, focusing on ACS. Special attention has been paid to the clinical studies of metabolomics and lipidomics in ACS, with an emphasis on ischemia/reperfusion injury.

## 1. Introduction

**Figure 1:**
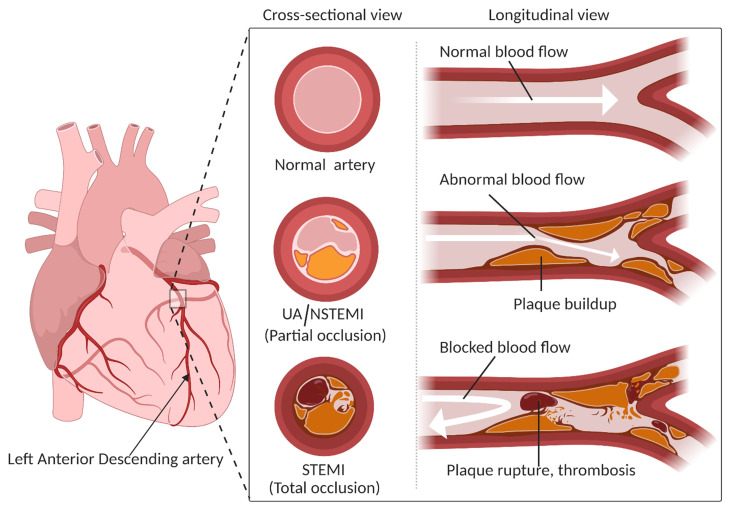
Classification of acute coronary syndromes. Acute coronary syndromes are categorized into unstable angina (UA), non-ST-segment elevation myocardial infarction (NSTEMI), and ST-segment elevation myocardial infarction (STEMI). A complete coronary artery occlusion due to thrombus formation results in STEMI, where the coronary blood flow is completely obstructed. A partial occlusion of the artery (blood flow is not entirely restricted) can result in NSTEMI or UA.

Coronary artery disease (CAD) continues to be a major public health concern with considerable morbidity and mortality in developed countries [1]. CAD includes chronic coronary artery disease (stable angina) and acute coronary syndrome (ACS), which almost invariably presents chest discomfort with or without dyspnea. It is estimated that in the United States alone, 720,000 people experience a new episode of ACS every year [2]. The costs and resource utilization associated with ACS also place an enormous economic burden on the healthcare system [2,3,4]. For instance, in a retrospective single-cohort study, it was found that the average one-year cost per patient associated with ACS was around 32,000 US dollars [4]. The term ACS refers to a spectrum of conditions in which myocardial ischemia or infarction develops due to acute occlusion of coronary blood flow to any part of the heart. The usual cause of acute occlusion is coronary artery thrombosis caused by rupture or erosion of a high-risk, lipid-laden, atheromatous plaque [5]. This sudden, reduced blood flow to the heart results in an imbalance between myocardial metabolic demands and blood supply, leading to myocardial ischemia, which is the hallmark of ACS [3,6]. The imbalance may also be caused by several other cardiac abnormalities, including coronary artery embolism, coronary spasm, coronary dissection, severe anemia, and calcific aortic valve stenosis [3]. Depending upon the range of ischemic state, location of the occlusion, cardiac biomarker levels (e.g., troponin), and ST-segment elevation on the electrocardiogram (ECG), ACS is mainly categorized into three types (Figure 1), namely unstable angina (UA), non-ST-segment elevation myocardial infarction (NSTEMI), and ST-segment elevation myocardial infarction (STEMI) [5]. Typically, a complete coronary artery occlusion leading to myocardial tissue injury and elevated cardiac troponin level results in STEMI. Partial occlusion or occlusion with collateral circulation may lead to NSTEMI or UA, depending on the presence or absence of cardiac troponin level, respectively [5,7]. Troponins are released into the bloodstream when there is any damage to the heart muscle [8]. Due to its high sensitivity and specificity, cardiac troponin measurement is an essential element in diagnosing and managing ACS.

The human heart is the most metabolically active organ, and under normal conditions, cardiac metabolism is tightly regulated by oxygen availability, substrate oxidation, hormonal and neurohumoral signals, among other factors [9]. The primary substrates for cardiac ATP production are lipids, carbohydrates, lactate, and glycogen [9]. Of these, lipids alone contribute to 60–90% of ATP production in the heart via oxidation of fatty acids [10]. Due to its exuberant use in the myocardium and its role in maintaining the myocardial cell structure and cardiac function, alterations in lipid metabolism contribute to many cardiovascular pathologies, including atherosclerosis, insulin resistance, hypertension, and type 2 diabetes mellitus [11]. A fall in oxygen level and substrate availability during myocardial ischemic conditions alters the dynamic equilibrium state of cardiac metabolism and leads to loss of homeostasis [12]. Hence, it is not surprising that ACS involves changes in cardiac metabolism. While some of the metabolic changes help the heart adjust to the altering substrate needs and physiological demands, most changes are maladaptive and initiate other cardiac abnormalities, including myocardial stunning, cell death, and contractile dysfunction [13]. Recent findings suggest that alterations in the cardiac metabolism alone can also perpetuate disturbances in systemic metabolism [14]. Hence, blood-derived plasma or serum metabolic profile could provide insights into the pathophysiological processes happening within the heart in the event of an ACS.

**Figure 2:**
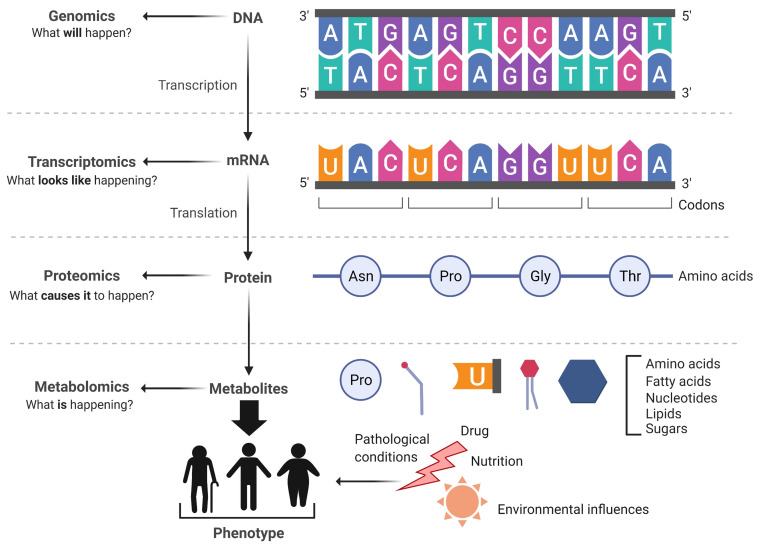
The ‘omics cascade’. It depicts the directional flow of biological information from genes to metabolites. Metabolomics is at the end of the cascade and is closer to the phenotype of an organism than proteomics or genomics.

Metabolomics is the new entrant in the ‘omics’ cascade in the systems biology (genomics, transcriptomics, and proteomics) approach (Figure 2). It involves high-throughput identification and quantification of small chemical compounds (<1000 to 1500 Da), commonly known as metabolites, present in a variety of biological system such as a cell, an organism, or biological fluids [15,16]. The complete set of these small-molecule metabolites and their interactions within the biological system are known as the metabolome. The metabolome includes endogenous (e.g., amino acids, fatty acids, sugars, carbohydrates, vitamins, lipids, and their derivatives) as well as exogenous (e.g., pollutants, pharmaceuticals, food additives, xenobiotics) compounds. Lipidomics is a novel subdivision of metabolomics, dedicated to the detailed analysis of complex lipid mixtures found in biological materials [17]. The metabolome composition is inherently dynamic and flexible due to its continuous interaction within the biological system and also with the outside environment, including the effects of drugs, nutrition, lifestyle, or therapeutics [18]. Hence, any perturbations in metabolite levels are a true reflection of the phenotype and function of the developmental or pathological state of the biological system. Recent advancements in ‘omics’ technology platforms, particularly genomics and proteomics technologies, have enabled us to assess the changes within the genome and the proteome in cardiovascular diseases (CVD), including ACS. Metabolomics and lipidomics are required to bridge the knowledge gap between phenotype and metabolic abnormalities in CVD. 

Given that ACS is a multifactorial phenomenon, it requires a global assessment of molecular/cellular pathological functions from both untargeted and targeted approaches. Recognizing this, the American Heart Association recently released a scientific statement highlighting the potential impact of metabolomics in cardiovascular health and disease [19]. Thus, metabolomics and lipidomics are now evolving as essential tools in cardiovascular research (i) to gain a better understanding of the mechanisms underlying CVD, (ii) to determine the clinically relevant metabolomic perturbations, and (iii) to identify novel biomarkers involved in various cardiovascular disease conditions.

## 2. Analytical Tools in Metabolomics

Though metabolomics research is considered a relatively new field, the initial report of screening metabolites in body fluids for diagnostic purposes can be traced back to ancient China (1500–2000 BC), where traditional Chinese physicians used ants to diagnose diabetes by evaluating the smell, taste, and color of urine samples from patients [20]. However, the pioneering work carried out by the laboratories of Robinson’s and Pauling’s research groups in the early 1970s [21,22,23] laid the foundation for a new era in metabolomics research. They successfully quantitated metabolites from various biofluids, including human blood and urine vapor, by applying the gas chromatography coupled with mass spectrometry (GC/MS) technique. The first printed reference to the word ‘metabolic profile’ appeared in the literature in 1971 [24,25], when Horning and Horning used the GC/MS platform to profile different human metabolic products, including sugars, alcohols, and drug metabolites from blood and urine samples. Since these initial efforts, several investigators have contributed in parallel to develop this naive concept into a format that is on par with the other established ‘omics’ approaches, namely genomics and proteomics. The word ‘metabolome’ was first used by Oliver Fiehn, a key metabolomics researcher, in 1998 [26] to denote the changes in relative concentrations of metabolites due to the deletion or overexpression of a gene. Due to the metabolome’s vast complexity and dynamic nature, no single instrument platform currently available can simultaneously analyze all the metabolites present in a biological sample. For example, within lipids alone, diverse lipid classes and lipid molecular species are categorized based on their structural and chemical properties. Moreover, while some lipids such as cholesteryl esters and triglycerides are usually present in high concentrations (1–10 mmol/L) in samples such as plasma, others such as eicosanoids derived from arachidonic acid are present only in trace amounts (1–10 pmol/L) [27]. Numerous analytical platforms with varying degrees of specificity and sensitivity have been developed in the last two decades to measure specific portions of the complex metabolome. Presently, nuclear magnetic resonance (NMR) spectroscopy and mass spectrometry (MS), coupled with gas or liquid chromatography (GC/MS or LC/MS, respectively), are the most widely used analytical tools in metabolomics investigations [15]. In addition, analytical platforms such as MS coupled with capillary electrophoresis (CE/MS), shotgun lipidomics, and direct MS infusion methods are also used to fully understand the broad spectrum of metabolites [15,28]. For instance, while NMR makes the study of different isomers possible, GC/MS or LC/MS can analyze a large variety of molecules with high sensitivity, and CE/MS can provide data of high resolution. Table 1 summarizes the main advantages and disadvantages of using MS and NMR techniques.

### 2.1. NMR Spectroscopy

NMR spectroscopy is one of the most prevalent techniques used in metabolomics research for structural elucidation of both pure compounds and mixtures. This technique relies on the magnetic spin properties (nuclear magnetic moment and angular momentum) of protons and neutrons in the nucleus of an atom to provide information on molecular structures. It is based on the principle that any nucleus possessing a (non-zero) nuclear spin can release characteristic radiofrequency waves when placed in an external magnetic field. The resonance frequency of the released energy is affected by the chemical microenvironment of these atoms and the coupling effect with nearby nuclei. The resulting frequencies are recorded as chemical shifts, and their patterns can be used for compound identification [15]. The most commonly used nuclei for analysis are ^1^H, ^13^C, and ^31^P isotopes. The proton NMR spectroscopy (^1^H-NMR) has the advantage of having greater relative sensitivity and a short acquisition time. For this reason, ^1^H-NMR is widely used to identify and quantify a large number of small molecules coexisting in biological material, such as tissues, whole cells, and biofluids. The major disadvantage of traditional one-dimensional (1D) ^1^H-NMR is that the spectrum became very complex for larger, more complex molecules, making interpretation difficult as most of the signals overlap heavily. However, two-dimensional (2D) NMR helps circumvent this challenge by resolving signals that usually overlap in ^1^H-NMR. In short, 2D-NMR provides extra information about a molecule compared to traditional ^1^H-NMR spectra, thereby making spectral interpretation easy. Current practice requires 1D ^1^H-NMR to complement 2D-NMR or mass spectrometer for molecular identity confirmation. Carbon-13 NMR spectroscopy (^13^C-NMR) and phosphorous-31 NMR spectroscopy (^31^P-NMR) predominantly find applications in cellular energetics, particularly in tracking cellular changes in cardiac metabolism under normoxic and ischemic conditions [29,30,31]. 

Table 2 shows that in ACS metabolomics studies, ^1^H-NMR spectroscopy is the most used NRM technique for analyzing biofluids, including plasma, serum, and urine. ^1^H-NMR spectroscopy is routinely used to analyze several low-molecular-weight molecules, including branched-chain amino acids (e.g., valine, isoleucine) and ketone bodies from plasma and serum to find biomarkers for ACS [32,33,34]. In a study looking at potential candidate biomarkers for UA from plasma samples, ^1^H-NMR spectroscopy identified ten steroid metabolites belonging to the steroid hormone biosynthesis pathway, including one mineralocorticoid (deoxycorticosterone) and nine sex hormone metabolites (e.g., estradiol, 2-hydroxyestradiol) [35]. Additionally, NMR has become the primary tool for analyzing urinary metabolites. In a study involving UA patients [36], ^1^H-NMR analysis could successfully identify and quantitate waste metabolites such as trimethylamine-N-oxide (TMAO) and trimethylamine (TMA) from urine specimens, in addition to short-chain fatty acids (e.g., 3-hydroxybutyrate), organic acids (e.g., indol-3-acetate, methylmalonate), and amino acids (e.g., lysine, proline).

The main advantages of NMR are its non-destructive nature and higher reproducibility [37,38]. However, it is often limited by its requirement of a larger sample amount (2–50 mg) and therefore reduced sensitivity for low abundant compounds [39]. An early metabolomics study showed that 1H-NMR spectroscopy could correctly diagnose the presence of CAD and assess its severity [40]. Other applications of NMR-based metabolic profiling in ACS mainly include analysis of both urine [36] and serum [33,34] metabolites for unstable angina pectoris disease, investigating the serum metabolic characteristics of acute myocardial infarction (AMI) patients in comparison with those of chest pain controls [41], and deciphering the metabolomic fingerprint of coronary blood in STEMI patients [42]. 

### 2.2. Mass Spectrometry

Mass spectrometry (MS) provides accurate weight measurements of one or more molecules within a sample of interest. MS separates the molecules based on their specific mass-to-charge ratio (*m/z*) by converting them into ions in the gas phase [72]. The separated ions are then sorted according to their acceleration and deflection in an external electromagnetic field. The final output is presented as the relative abundance of each ion as an *m/z* spectrum. MS is extensively used in the field due to its wide dynamic range, speed, high sensitivity, and the ability to identify and quantify more metabolites in a single measurement relative to NMR. 

The MS essentially has three main components: (1) the ion source, where the sample is ionized, (2) the mass analyzer, where the ions are separated according to their mass-to-charge (*m/z*) values, and (3) the detector, which provides a count of the intensity of separated ions [73]. The most commonly used mass analyzers are Time-of-Flight (TOF), magnetic sector, and quadrupole, each with its own set of strengths and limitations [74]. Time-of-Flight, as the name implies, uses a flight tube of known length, where the ions are separated based on the flight time (time taken for the ions to travel through the flight tube). TOF MS consists of a pulsed ion source and therefore is best suited with ionization methods that ionize molecules in pulses, such as laser ionization. TOF systems have an excellent mass range and are generally utilized for high-resolution MS. Unlike TOF systems, magnetic sector mass analyzers use a magnetic field to sort ions of different mass-to-charge ratios. The key advantages of magnetic sector mass analyzers are their high sensitivity and high resolution. As its name suggests, quadrupole consists of four parallel cylindrical or hyperbolic rods. The opposite rods are connected electrically inside a vacuum chamber. By changing the electrical potential, ions with different *m/z* values can be ‘filtered’ through the quadrupole to the detector one after another. The quadrupole mass analyzers are usually compact and have good scan speed, durability, and reliability. However, they usually have a limited mass range.

MS uses various ionization methods for mass analysis. The classic methods include matrix-assisted laser desorption ionization (MALDI) and electrospray ionization (ESI), in addition to other methods, such as electron impact (EI) ionization, chemical ionization, and atmospheric pressure chemical ionization (APCI) [75]. ESI is based on the evaporation of charged droplets and is notable for being the softest ionization method, allowing for generating ions with multiple charges with great sensitivity and no matrix interference [76]. However, some of the pitfalls of ESI include incompatibility with salts, complex mixtures, and impure samples. On the other hand, MALDI functions by proton absorption and transfer and can ionize metabolites with much higher masses (up to 300,000 Da). MALDI is also compatible with both salts in millimolar concentration as well as complex samples. One disadvantage of using MALDI is the potential for the degeneration of metabolites due to the acidic matrix or photogeneration via laser ionization. Additionally, MALDI is also not suitable to analyze low-molecular-weight compounds (<1000 *m/z*) because of the matrix background interferences in this mass range [77]. Overall, it has been shown that using multiple ionization approaches with mass spectrometry can enhance the detection of global metabolome for complex samples [78].

A separation technique in a time dimension (GC, LC, or CE) is often employed before MS analysis to isolate the individual components from complex samples containing hundreds to thousands of small molecules [79]. The chromatographic separation techniques use the physicochemical properties of the compounds in a sample, such as polarity, size, and presence of double bonds, to separate the molecules inside the medium [80]. Hence, combinations of separation and MS, usually either GC/MS or LC/MS, have become the preferred analytical choice for small-molecule analysis from complex biological samples. The application of MS-based metabolic fingerprinting in ACS mainly includes investigating biomarkers for ACS [68,69,70,71], characterizing the metabolic difference between different types of ACS [64,65,67], and identifying the molecular differences between patients with ACS and healthy controls [51,62].

For minimally complex samples such as synthetic peptides or pure compounds, direct infusion mass spectrometry (DIMS) or shotgun lipidomics (for lipid samples) are used. The samples are introduced directly into the MS without prior chromatographic separation. Though suited for high-throughput metabolomics analysis, DIMS often suffers from the matrix effect and ion suppression since all the sample components are infused simultaneously [81,82]. Direct infusion also leads to ion source contamination, which usually takes a long time to recover. Recently introduced chip-based direct-infusion nano-electrospray interfaces were successful in resolving this problem to a great extent [83]. Due to the lack of chromatographic separation, DIMS also cannot separate isomeric compounds. One approach to achieving isomeric separation is incorporating ion mobility spectrometry (IMS) as the separation process before MS [84]. Another drawback of the DIMS system is that multiple ions of the same molecule, such as its molecular ions, adducts, and in-source fragments, can be present in the mass spectrum, thus making interpretation of the data very difficult. 

Compared to NMR, MS is the method of choice for global metabolite profiling and identifying unknown compounds within samples. MS can detect various metabolite classes (polar, non-polar, and neutral) depending on the choice of ionization mode (positive/negative). For instance, positive ESI mode works well with medium-sized polar molecules, whereas negative ESI mode is suitable for carbohydrates and organic acids. It is worth mentioning that all clinical studies of lipidomics in ACS mentioned in Table 3 were carried out using the LC/MS platform. Additionally, MS also provides an excellent analytical platform for profiling the molecular composition of lipoprotein complexes. For example, multiple clinical studies [85,86,87] employed the LC/MS platform to highlight the changes in lipidome composition of HDL (high-density lipoprotein) during ACS pathogenesis. 

**Figure 3:**
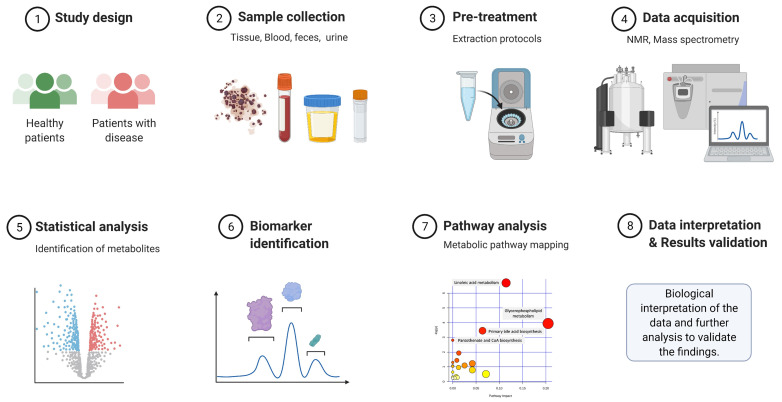
A standard workflow for untargeted metabolomics. After sample collection and extraction, the samples are analyzed by NMR spectroscopy or mass spectrometry. The raw data are then analyzed using appropriate software followed by statistical analysis to identify metabolites of interest or potential candidate biomarkers. Adapted from “Untargeted Metabolomics for Discovery of Disease Biomarkers”, by BioRender.com (2021). Retrieved from https://app.biorender.com/biorender-templates on August 11, 2021.

NMR and MS primarily use two strategies to conduct metabolomics and lipidomics studies, i.e., untargeted, and targeted approaches [101,102]. The untargeted approach, also called open profiling, aims to analyze the most comprehensive set of metabolites from biological samples and is non-selective and non-discriminative towards metabolites screening. It is mainly used for the discovery of novel biomarkers or to generate a specific hypothesis. Usually, no pre-existing knowledge about the metabolic profile of the sample is required for this type of analysis. The untargeted approach usually compares metabolite levels between different groups (e.g., healthy control vs. disease) under similar conditions [101,103]. A schematic showing the different steps in untargeted metabolomics is shown in Figure 3.

On the other hand, the targeted approach or closed profiling focuses on a limited number of known metabolites, such as 10–20 lipids, and seeks to quantify them. A hypothesis is being tested in this approach, and often the sample preparation and analytical techniques are more sophisticated. In the case of ACS, the most used approach so far has been untargeted metabolomics to differentiate the ACS profile from healthy controls. However, most ACS studies complement their untargeted analysis by performing a targeted analysis of those compounds that exhibited a significant difference between various groups for further quantitation and identity confirmation using the suitable internal standards. In a study to understand the underlying mechanisms associated with CAD progression, Zhang and colleagues initially performed an untargeted analysis of metabolites in plasma of 2324 patients who underwent coronary angiography [61]. They identified a total of 36 differential metabolites across different CAD types, including N-acetylneuraminic acid (a ligand for many hormones and lectins), whose levels were elevated in plasma during CAD progression. Subsequently, a targeted quantification was performed using isotope-labeled N-acetylneuraminic acid to confirm its vital role in CAD progression.

## 3. Pre-Analytical Considerations in Metabolomics Studies

The analytical quality of both untargeted and targeted metabolomics approaches is mainly dependent on the various pre-analytical steps involved in sample handling, sample collection, centrifugation, aliquoting, transportation, freezing, and storage [104]. Even minor discrepancies in any of these pre-analytical factors can greatly influence metabolomic assessments. Discrepancies may include, but are not limited to, choice of anticoagulants or preservatives added to the sample specimen, time delay, and storage temperature during blood preprocessing [105], diet and time of sampling in urinalysis [106,107], metabolite degradation or aggregation induced by air oxidation and light exposure [108,109], number of freeze–thaw cycles before aliquoting biological fluids [110], and time delay in transportation and sample storage [111]. Therefore, knowledge about different sample collection procedures and preparation protocols is essential to ensure reliable and reproducible results in a metabolomics study. A variety of biological fluids, including plasma, serum, urine, saliva, cerebrospinal fluid, bronchoalveolar lavage fluid, and cell and tissue extracts, are used in metabolomics studies depending upon the biological question under study and the pathophysiological nature of the disease process [112].

### 3.1. Serum vs. Plasma

Notably, most of the metabolomics studies in ACS have been performed using either plasma or serum as the sample source [33,34,69,70,71]. The general theme of most of these plasma- or serum-based studies is to find biomarkers associated with ACS and its subtypes, compared to healthy controls. Though both plasma and serum matrices provide an excellent functional readout of an organism’s metabolic activity, the question regarding which is more suitable is still highly debatable. Both plasma and serum are derived from the liquid portion of the blood. The serum is extracted after the blood has clotted, and it is obtained by centrifugation after clotting. For plasma, the blood is not coagulated and is obtained by adding an appropriate anticoagulant (heparin, citrate, or Ethylenediaminetetraacetic acid (EDTA)) to the whole blood, followed by centrifugation and aliquoting [112]. Typically, plasma makes up to 55% of the total volume of blood, and it contains dissolved proteins, clotting factors, salts, lipids, and other suspended materials in water. The serum is similar to plasma, except it lacks fibrinogen (clotting factor). Contrary to serum, plasma has the advantage that it can be immediately placed on ice, thereby avoiding the possible loss of labile metabolites due to enzymatic conversion or other degradation processes during clot formation at room temperature [112]. Several studies have tested the role of pre-analytical factors on the metabolic composition of serum and plasma [113,114,115]. Teahan et al. showed that variations in certain pre-analytical factors such as clotting time and temperature, the presence/absence of anticoagulant, and storage conditions including repeated freeze−thaw cycles, can introduce bias into metabolic data [115]. Additionally, recent data suggest that it is not advisable to combine serum samples exposed to different clotting procedures (e.g., thrombin vs. silicate-enhanced) or different clotting times into a single sample set for biomarker analysis, as coagulation and associated processes can alter metabolite concentrations [116]. This limitation can be highly challenging, particularly in multicentric clinical studies involving humans, where samples are collected from different hospital sites and transported to a centralized facility for further processing and analysis. 

Besides assessing various pre-analytical factors, numerous studies have also investigated the difference in metabolic profile generated by serum and plasma samples [116,117,118]. In a study comparing serum and plasma metabolic profiles [116], it was found out that 46% out of the 216 identified metabolites were significantly different between the two, and except for three (methionine, C2:0-, and C3:0-carnitine), the levels of all other metabolites were higher in serum. In line with this, in another metabolomics study comprising 377 individuals [118], it was reported that although the reproducibility was slightly better in plasma than serum, the concentrations of analytes were generally higher in serum, with an average relative difference of 11.7%. In another study involving 29 small-cell lung cancer patients [117], it was shown that neither fluid is superior to the other, and the two metabolomes were markedly similar in terms of reproducibility, specificity, and metabolic coverage. These studies suggest that even though both biofluids are comparable in terms of analytical throughput, serum may provide better sensitivity than plasma.

### 3.2. Polar vs. Non-Polar Metabolites

Due to the enormous diversity in the physicochemical properties of metabolites, especially polar (including amino acids, nucleic acids, and sugars) and non-polar (including fatty acids and other lipids) metabolite classes, no ideal extraction protocol exists that can extract both polar and non-polar metabolites from the same sample. Accordingly, a comprehensive analysis of the entire metabolome in a biological sample requires multiple extraction strategies to cover different metabolite classes. Hence, multiple aliquots of the same sample with specific extraction procedures, such as using a modified Bligh–Dyer protocol for extracting polar metabolites [119], are required to obtain extensive coverage of the entire cellular metabolome, which in turn facilitates the need of a larger sample amount. In studies where sample amount is a limiting factor, this approach might not be feasible. Of late, efforts have been made to develop extraction protocols that can extract both polar and non-polar analytes from the same sample [120]. Selecting the proper extraction protocol is critical in all metabolomics studies as the metabolite extraction step directly affects all downstream steps of metabolomics data analysis.

## 4. Extraction Procedures for Metabolomics

The reliability and accuracy of metabolic data also depend upon the sample preparation strategy. Ideally, the sample processing should be fast, easy, minimal, and tailored to the analytical scheme. Common approaches include homogenization, dialysis, fractionation, extraction, distillation, centrifugation, and concentration. Critically, these approaches should be compatible with the nature of the biological matrix, chemical/physical properties of the analyte of interest, and the final detection technology. For example, in a typical LC/MS platform, sample preparation is usually the most time-consuming and error-prone step of the chromatographic assay. The inherent ‘matrix effect’ on different samples such as plasma, serum, urine, and tissue lysate, presents significant analytical challenges during LC/MS analysis [121]. Matrix effects are caused by co-eluting endogenous components and preservative agents in the same matrix [122], which often lead to material buildup on the analytical column and ion source in LC and MS, respectively. This can cause ion suppression or enhancement, drift in chromatographic response, increased or reduced analytical signal, reduced column life, and frequent MS cleaning [123]. Cumulatively, these issues may compromise analytical accuracy and increase the total analysis time, in addition to significantly affecting the cost of MS analysis [121]. Therefore, thoughtful selection and optimizing sample preparation procedures are essential to minimize variability and improve analytical performance in metabolomics studies.

The three most popular sample preparation techniques in metabolomics analysis include protein precipitation (PPT), liquid–liquid extraction (LLE), and solid-phase extraction (SPE) [124,125]. PPT generally begins with adding organic solvents such as methanol, acetonitrile, or a combination thereof to the sample, followed by agitation and centrifugation [126]. Its ‘nonselective’ nature makes it well-suited for global metabolomics analysis. Adding an ice-cold organic solvent is beneficial as it may improve the efficiency of protein removal and prevent metabolite degradation as the sample warms up from its stored frozen state [127]. While agitation increases the protein accumulation rate, centrifugation helps separate the supernatant holding analytes from the protein pellets [128]. Though this method provides high metabolite coverage, PPT is often time-consuming, particularly while dealing with hundreds of samples manually, as in large-scale epidemiological studies. Robotic systems capable of performing automated protein precipitation such as membrane-based protein precipitation filter plates have been developed recently to address this issue [124,129,130].

Another classic method used for the qualitative/quantitative identification of metabolites is biphasic LLE. LLE utilizes two immiscible liquids for the extraction of analytes. In LLE, the analyte is differentially distributed between the aqueous matrix and water-immiscible organic solvent [124]. Traditionally, LLE is most preferred for comprehensive lipid analysis [131] (i.e., global lipidomics). Apart from enriching analytes of interest (here, lipids) and improving signal-to-noise ratios, any extraction protocol used in lipidomics should also remove any non-lipid compounds. Being insoluble in water, lipids are extracted from a biological matrix, such as blood plasma or tissue, using organic solvents. The most widely used method for isolating lipids from biological samples was devised by Folch et al. more than 60 years ago using 2:1 chloroform/methanol (*v/v*) as the solvent mixture [132]. In this two-phase LLE procedure, most lipids are dispersed into the lower chloroform phase, and are clearly separated from the upper methanolic phase holding non-lipid substances. To enhance recovery, Bligh and Dyer later revised Folch’s method by combining chloroform, methanol, and water [133]. The disadvantage of these two methods is that the recovery of the lower lipid fraction is often cumbersome, resulting in contamination of the isolate and blockage of the analytical column. In 2008, Matyash and co-workers [134] showed that an extraction procedure using methyl-*tert*-butyl ether (MTBE) could solve this problem as the lipid-containing organic layer settles at the top during phase separation, thereby enabling a much cleaner lipid extraction. More recently, a chloroform-free one-phase lipid extraction protocol based on a mixture of butanol and methanol (BUME) was described by Löfgren and coworkers [135]. This novel approach is rapid and shows similar extraction efficiency compared to the Folch and the Bligh and Dyer procedures.

In SPE, samples are loaded onto a solid sorbent held primarily on a cartridge device (SPE cartridge). The analyte of interest present in samples is selectively retained by the sorbent material [136]. The retained analyte is then eluted with a suitable solvent [137]. Different SPE types include reverse, normal, anion, cation, and mixed-mode sorbents [124]. Due to its highly selective nature, SPE is not recommended for global metabolomics profiling [138]. It is mainly used to concentrate analytes present at low levels and purify analytes from matrix interferences. There exist contradictory reports regarding the analytical reproducibility using the SPE procedure. In a study comparing PPT and SPE procedures, SPE showed improved repeatability compared to PPT for human plasma metabolic profiling [139]. However, in another similar study evaluating different human plasma preparation protocols, the metabolome coverage and repeatability of the SPE procedure are reported to be lower than PPT protocols [126]. Once an extraction procedure is selected, caution should be taken to ensure that the same procedure is repeated consistently throughout the entire study. For large-scale cohort studies, maintaining this consistency presents a real challenge. Inconsistency in extraction procedures is a source of variation in the dataset and may compromise the robustness and accuracy of the results.

## 5. Data Processing in Metabolomics

As with the genomics platform, metabolomics studies generate a large amount of multidimensional, non-linear, and non-normal data [140]. Due to its sheer complexity, the metabolomics data-analytical approach relies heavily on advanced computational approaches. Many instrument-specific and open-source software solutions are available, such as MetaboAnalyst [141] and XCMS [142], that can perform the critically important steps in metabolomics data analysis, such as run alignment, peak picking, data preprocessing (e.g., deconvolution, scaling, and normalization), annotation, and compound identification. However, selecting a suitable approach or software is often confusing and depends upon the separation technique, detection mode, and output file format (e.g., mzXML, mzML, NetCDF) generated by each instrument. Additionally, there remains little accord or harmonization among different software in handling various cross-vendor file formats. Recently, much effort has been made to address this issue, and new software platforms such as Progenesis QI [143] have been developed, that are compatible with a wide variety of instruments and can perform platform-independent metabolomics data analysis.

### 5.1. Handling Unwanted Variances in Metabolomics Data

Analytical and biological variability issues are critical in human metabolomics studies. If these unwanted variations are not accounted for, it may affect the statistical power of detecting metabolites that are characteristic of, for example, a disease state such as ACS. Laboratory procedures or technological platforms primarily introduce analytical variability. Typical examples include matrix effects, temperature changes, solvent pH changes, sample degradation over time in extended sample runs spanning weeks or months, fluctuation in instrumental sensitivity, and personal errors [144]. One way to combat these analytical errors is by employing suitable sample normalization strategies, such as data-driven normalizations, internal standards-based normalization, or quality control (QC)-based normalization [145]. These strategies help to standardize metabolite abundances before statistical analyses and improve the quality of metabolomics data. Of these, QC-based normalization approaches are gaining more popularity of late [144,146]. Several QC-based normalization algorithms have been developed in recent times, including batch-ratio [147], LOESS [148], and SERFF [145]. The QC samples are injected at regular intervals along with the study samples in every batch. Ideally, the QC sample should have the same matrix composition as that of study samples and is usually obtained by pooling multiple aliquots. After QC-based batch correction, those metabolites which exhibited poor repeatability across QC samples were often removed based on specific cut-off criteria to assure an expected level of data quality. For example, after batch correction, only those metabolites satisfying the cut-off criteria were often retained for subsequent data analysis [149], such as (1) metabolites present in >50% of QC samples, and (2) metabolites with a coefficient of variation for QC samples <20%. 

Though the above-mentioned approaches can largely eliminate unwanted analytical variations from the dataset, the inherent biological variability induced by factors such as diet, ethnicity, physical activity, circadian rhythm, and medications still poses a significant challenge in interpreting the real physiological variations due to disease status or interventions [150]. To overcome these challenges, several strategies have been proposed, including cross-validation of results using an appropriate proportion of test and validation sets (e.g., 30%, the ‘test set’), validation of identified biomarkers using large cohorts of multi-centric samples (validation set), reporting results in relative amounts rather than absolute amounts of metabolites, and finally, validation of candidate biomarkers based on comparison to isotopically labeled internal standards. Current practice involves using different software for different tasks in the metabolomics workflow, such as one for peak picking, another one for normalization, and another one for metabolite annotations. This is a very tedious process and a time-consuming one. As we move forward, particular emphasis should be given to developing software that can automate this process from aligning runs to peak picking to batch correction to metabolite annotations, which may become “current practices” in the immediate future.

## 6. Metabolomics in ACS

Recently, there has been a growing appreciation for metabolomics as a promising approach for investigating cardiovascular diseases, including CAD to allow for better mechanistic understanding and biomarker discovery. The studies in the 2000s by Sabatine and colleagues initially demonstrated the application of a blood-based metabolomics platform to identify markers associated with myocardial injury. In a study comprising 36 subjects who underwent exercise stress testing (inducible ischemia), Sabatine et al. showed that myocardial ischemia is characterized by significant changes in the circulating levels of multiple metabolites, including lactic acid (final product of glycolysis) [151]. The pathway analysis identified a key role for the TCA cycle (regulator of oxidative phosphorylation) during myocardial ischemia. Moreover, they identified a panel of six metabolites, including citric acid, which could accurately stratify patients with myocardial ischemia from control subjects. Following this study, in a human model of planned myocardial infarction, via alcohol septal ablation, the same group identified metabolic changes as early as 10 minutes after myocardial injury [152]. They reported that perturbations in pyrimidine metabolism, the TCA cycle, and the pentose phosphate pathway were associated with myocardial injury. Importantly, these perturbations were observed in both coronary sinus and peripheral blood and were further validated in an independent clinical cohort.

A comprehensive overview of the clinical studies of metabolomics in ACS is provided in Table 2. Only those studies which explicitly discuss any of the ACS subtypes were included in the table. As evident from Table 2, blood-derived plasma and serum from veins are the most preferred biological fluids, and LC/MS is the favored technique being used in these studies. However, the sampling time varies greatly between these studies. First-morning collection, collection after overnight fasting, spot sampling at the time of admission (non-fasting), and timed collection at different sampling time points are the various sampling modes employed in these studies. The ideal sampling time is when the rate of metabolic flow, or flux, is constant, i.e., a steady metabolic state. However, ACS always involves ‘metabolic shifts’, and thus there is no steady metabolic state available in ACS events. Under these circumstances, time-series analyses could provide a better insight into the molecules and pathways with clinical relevance.

The initial clinical studies of metabolomics in ACS made use of GC/MS to identify biomarkers for the early diagnosis of ACS. Utilizing GC/MS, Vallejo et al. and Laborde et al. elucidated the metabolic differences in plasma of non-ST-elevation ACS patients relative to healthy subjects [68,71]. These studies collectively identified tricarboxylic acid (TCA) cycle intermediates along with 4-hydroxyproline, tryptophan, 3-OH-butyric acid, and 2-OH-butyric acid as key players in ACS pathophysiology. Subsequent studies employed NMR, LC/MS, and CE/MS approaches, shifting focus from the broad spectrum of ACS towards various ACS subtypes. Using CE/MS and hydrophilic interaction chromatography/MS-targeted analysis, Naz et al. found increased acylcarnitines (associated with defective mitochondrial β-oxidation) and amino acids (involved in myocardial energy metabolism) levels in STEMI patients compared to NSTEMI patients [67]. Another study used a combination of different metabolomics approaches, including GC/MS and H-NMR, and confirmed the presence of elevated hydrogen sulfide (an endogenous gasotransmitter) levels in STEMI patients compared to UA patients [63]. In a large study comprising 2324 patients from 4 independent centers [64], Fan et al. evaluated the diagnostic value of plasma metabolomics to characterize different types of CAD. Based on CAD severity, patients were divided into five groups, of those with the normal coronary artery, nonobstructive coronary atherosclerosis, stable angina, UA, and AMI. They found 89 differential metabolites across different CAD types. Additionally, they identified glycerophospholipid metabolism, amino acids, acylcarnitines, TCA cycle, and bile acid biosynthesis as the main metabolic pathways associated with CAD progression. Importantly, these findings were replicated in a validation cohort.

Metabolomics has also been used to explore biomarkers predictive of adverse cardiovascular events following ACS. For instance, Du et al. performed LC/MS analysis of 26 amino acids in a cohort of 138 STEMI patients with acute heart failure to find metabolites predictive of adverse cardiovascular events [58]. They found that elevated plasma branched-chain amino acids (BCAA) levels on admission are associated with adverse cardiovascular events. In another study comprising 978 patients [54], Vignoli et al. used NMR-based metabolomics to identify prognostic markers of two-year mortality after AMI. They showed that elevated levels of amino acids including mannose, formate, acetone, proline, creatinine, acetate, and 3-hydroxybutyrate were associated with mortality following AMI. Another study used an untargeted LC/MS approach and showed that six metabolic pathways, namely urea cycle, tyrosine, lysine, tryptophan, aspartate/asparagine, and carnitine shuttle, are associated with mortality in patients with CAD [47]. More recently, Chorell et al. showed that lysophospholipids (involved in inflammation and arteriosclerosis) are associated with future cardiovascular risk in STEMI and NSTEMI patients [44]. They reported that while STEMI is characterized by a higher ratio of lysophosphatidylcholine to lysophosphatidylethanolamine, NSTEMI is characterized by a lower ratio of these two lipids [44].

In recent times, lipid molecules and their associated pathways gained particular interest in the setting of ACS. In 2018, Wang et al. demonstrated that in addition to TCA cycle intermediates and amino acid metabolism, other lipid-associated pathways, including fatty acid metabolism and fatty acid β-oxidation, also play important roles in ACS [56]. During the same time, Goulart et al. showed that the most perturbed metabolites associated with STEMI were primarily lipid species, including phosphatidylcholines, lysophosphatidylcholines, and sphingomyelins [55]. These results underscore the need for comprehensive lipid profiling to provide insight into ACS pathogenesis.

One of the most consistent findings in these clinical studies has been the link between carnitine (short-chain and long-chain) and lysophosphatidylcholine (LPC) species with ACS. Carnitines play a critical role in transporting long-chain fatty acids from the cytoplasm into the mitochondria, where they undergo β-oxidation to produce energy. Accumulating evidence suggests that elevated levels of carnitines reflect impaired β-oxidation and mitochondrial dysfunction [153] and are associated with a wide variety of disorders, including type 2 diabetes [154], and cardiovascular diseases [155]. Since long-chain fatty acylcarnitines are produced from fatty acid metabolism and are primarily synthesized in the mitochondria, their levels indicate mitochondrial fatty acid oxidation [156]. On the other hand, LPC is a group of proinflammatory lipids, which is primarily derived from phosphatidylcholine (PC) by the enzymatic action of phospholipase A2 (PLA2). LPC has been linked to the pathogenesis of atherosclerosis and the progression of various diseases [157], including cardiovascular diseases, renal failure [158], ovarian cancer [159], and diabetes [160]. Among other properties, LPC also activates several signaling pathways, such as oxidative stress and inflammatory responses, contributing to endothelial cell injury in atherosclerosis and cardiovascular disorders [161].

## 7. Lipidomics in ACS

Apart from their primary role as the structural components of cells, lipids exert indispensable functionalities as cell signaling molecules and energy sources. Evidence from genomics studies and large randomized controlled trials has established the link between the dysregulated lipid metabolism and CAD progression, including ACS. The results from clinical trials of lipid-modifying therapy demonstrated that lowering the levels of serum lipids (especially cholesterol) reduces the risk of cardiovascular events [162]. Given the crucial role of lipids in regulating health and disease states, elucidating the lipid composition at the molecular and system level is essential to characterize the molecular basis of ACS. Therefore, there is a growing interest in lipidomics as a promising approach to reveal lipid alterations in ACS progression and to find new biomarkers for early ACS diagnosis. Table 3 summarizes the applications of lipidomics on ACS in clinical settings. From Table 3, it is evident that MS has been the dominating technique for lipid profiling, and blood-derived plasma is the most preferred biospecimen.

Traditional clinical lipid biomarkers for the development and progression of CAD, including elevated serum low-density lipoprotein (LDL), decreased high-density lipoprotein (HDL), or increased triglycerides levels, often fail to correctly distinguish ACS from stable coronary artery disease. Using plasma lipid profiling on 220 individuals, Meikle and colleagues showed that multivariate models incorporating both lipids and conventional risk factors could stratify unstable CAD from stable CAD patients with better accuracy than models with conventional risk factors alone [100]. The plasma levels of many lipids, including alkylphosphatidylcholine and phosphatidylcholine plasmalogen (these species are susceptible to oxidative stress), displayed a significant association with disease severity, suggesting their part in the onset and progression of ACS. In a report to characterize lipid species within lipoprotein particles, Meikle also reported that the levels of phospholipids, including lysophospholipids and plasmalogens, were significantly lower within the HDL of the ACS group relative to the CAD group [90]. In line with this finding, Sutter et al. and Rached et al. showed the contribution of alterations in the HDL lipidome to the disease severity of ACS [85,86]. Similarly, looking at 365 lipids, Lee et al. demonstrated that the levels of saturated lysophosphatidylcholine (LPC) species (16:0 and 18:0) were increased only in the HDL fraction of the ACS group, indicating an intermediate link between LPC species and progression of ACS [87]. In another study using a targeted lipidomics approach, Garcia et al. showed that the HDL2 subclass of ACS patients is enriched with oxidized fatty acids compared to non-ACS subjects, which may modulate platelet-dependent thrombotic risk [94]. Together, these studies demonstrate the ability of whole-plasma and lipoprotein-specific lipidomics for the early detection of ACS and discriminating stable CAD from ACS.

Recently, several studies have looked at the association between molecular lipid species and clinical outcomes in patients with ACS. In a study comprising 581 patients with ACS or stable CAD, Cheng et al. investigated the association of plasma lipids with 1-year clinical outcome [98]. They showed that plasma concentration of ceramides (involved in inflammation, membrane integrity, and apoptosis), particularly Cer(d18:1/16:0), is strongly associated with 1-year major adverse cardiac events (MACE) and plaque vulnerability, independent of statin usage and LDL levels. The prognostic value of these high-risk circulating ceramide species was further probed in the prospective ATHEROREMO cohort of 581 patients with stable angina pectoris or ACS and a median follow-up of 4.7 years [92]. Multivariable analyses showed that the circulating levels of Cer(d18:1/16:0), Cer(d18:1/20:0), Cer(d18:1/24:1), and their ratios were associated with adverse cardiac outcomes independent of the established clinical risk factors. A recent lipidomics analysis by Carvalho et al. employing paired tissue–plasma samples in human and animal models took these results a step further [95]. They showed that arterial and myocardial tissue ceramide levels also correlate with MACE in patients with AMI. Collectively, these data suggest a predictive role of plasma ceramide species in patients with ACS.

## 8. Metabolomics of Ischemia/Reperfusion Injury

**Figure 4:**
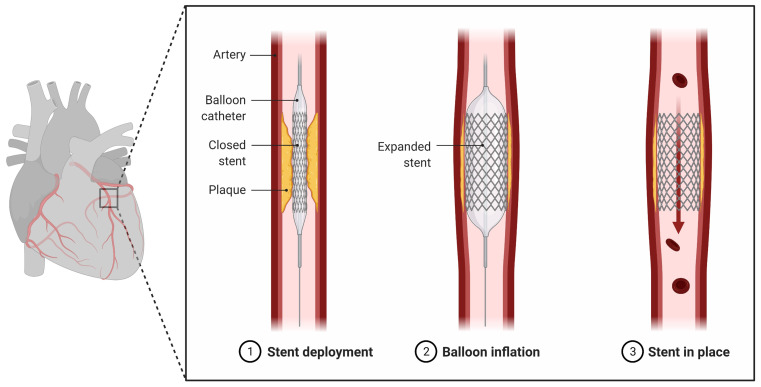
Percutaneous coronary intervention (PCI): PCI widens the blocked or narrow coronary arteries, thereby allowing reperfusion of the ischemic myocardium. Adapted from “Percutaneous Coronary Intervention”, by BioRender.com (2021). Retrieved from https://app.biorender.com/biorender-templates on August 11, 2021.

Timely myocardial reperfusion strategies employing fibrinolytic therapy or percutaneous coronary intervention (PCI) are the treatment of choice for acute STEMI patients. Besides salvaging viable cardiomyocytes from ischemic death, reperfusion profoundly limits the infarct size following a prolonged ischemic insult and improves the clinical outcome. However, pre-clinical and clinical data show that this sudden reintroduction of oxygen and nutrients during reperfusion by itself induces cardiomyocyte death, a phenomenon termed myocardial reperfusion injury [13,163]. Clinically, the largest effect of ischemia/reperfusion (I/R) injury is during PCI of STEMI patients. In STEMI patients, once the coronary artery is opened by PCI (Figure 4), it allows for reperfusion of the ischemic myocardium. Despite rapid and successful reperfusion, the mortality rate after an AMI is nearly 10% [164].

**Figure 5:**
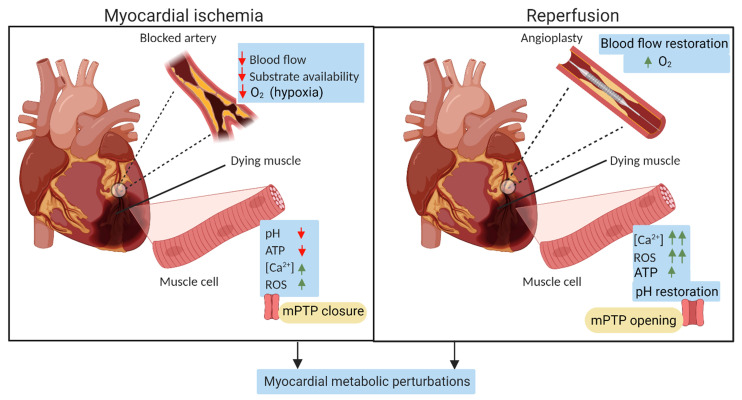
Myocardial ischemia/reperfusion (I/R) injury. Schematic showing the main events in myocardial I/R injury. Abbreviations: Ca^2+^, Calcium ion; ROS, reactive oxygen species; mPTP, mitochondrial permeability transition pore.

Low blood flow during acute ischemia switches cell metabolism to anaerobic metabolism, resulting in lactate accumulation and lowering of intracellular pH [165]. Additionally, there is a decrease in cellular ATP, which results in Ca^2+^ overload [165]. These processes disturb cell volume regulatory mechanisms, leading to disrupted cellular structure and cell lysis. Thus, it is essential to restore tissue oxygen supply. However, reperfusion leads to a sudden increase in the amount of oxygen available that causes an intense burst of mitochondrial reactive oxygen species (ROS), resulting in cellular dysfunction by modifying intracellular molecules [165]. Reperfusion also restores the physiological pH, which releases the inhibitory effect on mitochondrial permeability transition pores’ (mPTP) opening. Moreover, reperfusion results in intracellular Ca2+-overload due to the dysfunction of the sarcoplasmic reticulum. Reperfusion also initiates endoplasmic reticulum stress, a pro-inflammatory response, and pro-thrombogenic pathway activation in ischemic tissues [163,165]. The excess Ca^2+^ and enhanced ROS production trigger mPTP opening, resulting in ATP depletion and, ultimately, cellular death. A graphic indicating the main proponents of myocardial I/R injury is shown in Figure 5. In brief, reperfusion is associated with an additional injury that extends the ischemic damage. As a result of this puzzling problem between ischemic injury vs. reperfusion injury, there is significant interest in finding ways to protect against and treat the latter.

The ROS generation during reperfusion was previously thought of as a non-specific response to reoxygenation of ischemic tissue. However, a recent comparative in vivo metabolomic analysis in a mouse model of I/R injury has revealed that the TCA cycle intermediate, succinate accumulates during ischemia and the oxidation of succinate during reperfusion drives mitochondrial ROS accumulation and reperfusion injury [166]. A later study used a targeted LC/MS approach to analyze plasma metabolites in 115 STEMI patients undergoing PCI and revealed that myocardial succinate accumulation is an early marker of human I/R injury [60]. They reported an association between myocardial content of succinate and the magnitude of ischemic injury in STEMI patients. 

Recent metabolomics studies have also implicated the role of BCAAs in the development of I/R injury. In a metabolomics study utilizing a KO mouse model (2C-type ser/thr protein phosphatase (PP2Cm) deficient), Li et al. showed that impaired BCAA catabolism suppresses glucose uptake and worsens the I/R injury [167]. In line with this finding, our previous study in a clinical setting of human I/R injury identified BCAA (leucine, isoleucine, and valine) biosynthesis as one of the most perturbed metabolic pathways during early reperfusion [52]. Our study showed that lipids and their derivatives formed the bulk of the altered metabolome in the setting of reperfusion injury in STEMI patients [52]. We also identified a panel of three lipid molecules, namely pentadecanoic acid, 18:2 carnitine, and 18:2 lysophosphatidylcholine, that can determine the extent of I/R injury in STEMI patients after primary PCI.

A few studies have also explored the time-course changes in the circulating metabolic profile following reperfusion. Based on a time-series analysis before and after PCI comprising 40 STEMI patients, Feng et al. showed that the fatty acid content in the circulating blood gradually decreases with an increase in reperfusion time [93]. In another study monitoring the changes in eicosanoid profile before and after PCI in 20 STEMI patients, Zhang et al. found that the levels of some bioactive eicosanoids, including PGE2, PGD2, TXA2, and 20-HETE, were significantly decreased after PCI. Interestingly, these molecules are pro-inflammatory and are associated with platelet aggregation [168]. 

There are currently no therapeutic options for I/R injury in patients presenting with STEMI who undergo revascularization. Given the complex metabolic changes within the myocardium and hence within the circulation during I/R injury, it is necessary to identify the metabolic and lipidomic pathway(s) that impact clinical outcomes. There are many limitations to the current studies in the setting of I/R injury, including focusing on a single metabolite (e.g., succinate), a specific metabolite class (e.g., fatty acids or eicosanoids), a single pathway (e.g., BCAA metabolism), or having small sample sizes. Animal studies and planned strategies (e.g., inducible ischemia) are helpful to understand pathological mechanisms. Nevertheless, more clinical investigations incorporating time course analyses in large cohorts are needed to better understand the dynamic changes and metabolic pathways involved in human I/R injury.

## 9. Translational Metabolomics and Future Directions

Despite showing substantial potential for biomarker discovery and a more detailed understanding of pathogeneses of ACS, few findings from metabolomics studies have been translated into disease diagnostics and risk prediction. One main reason is the validity and reliability of new markers/clusters of metabolites [169]. The main challenge in biomarker validation is the difficulty in measuring subtle differences in metabolite fluctuations associated with different disease status or interventions. Additionally, as evident from Table 2 and Table 3, barring a few, most of the clinical metabolomics studies on ACS are small-scale preliminary-type studies performed with a limited sample size (*n* < 100), which lack the required statistical robustness and validity. We believe the only way to overcome this is to perform large clinical studies to allow for successful translation of metabolomics to clinics. In addition, as discussed in Section 5.1, the inter-individual variation induced by diet, genetic, and environmental exposures, coupled with technical variability, also diminishes metabolomics studies’ power to detect actual physiological variations associated with different ACS subtypes and, eventually, to provide clinical biomarkers [170]. One way to overcome this is by conducting follow-up metabolomics experiments using a large validation cohort consisting of diverse patient groups (multi-centric) with suitable control cohorts. However, large-scale multi-cohort studies are usually laborious, expensive, and time-consuming. Once validated appropriately, there are increased prospects of translating these biomarkers towards clinics and diagnostic centers. Another potential challenge in translational metabolomics is that most of the metabolomics studies in ACS published thus far were focused on generating data and interpreting them without delving into the mechanistic link underlying the association between the identified metabolites and ACS pathogeneses [171]. Finding the relation between candidate biomarkers and their biological role is the next important step after biomarker discovery. This can be carried out by employing suitable in vitro and animal model experiments. Importantly, in the future, the metabolomics community should work in tandem with other omics communities such as genomics and transcriptomics to gain more insights on cellular processes represented by a candidate marker [172]. This will bring us closer to a mechanistic understanding of various physiological and pathophysiological conditions associated with various disease states, including ACS.

## 10. Conclusions

Over the past decade, metabolomics has become a powerful investigative tool to elucidate the underlying metabolic mechanisms of cardiovascular diseases. Most of the metabolomics studies in ACS have focused on biomarker identification to differentiate ACS from healthy controls, to differentiate ACS subtypes (UA, NSTEMI, and STEMI), and to find predictive molecules of mortality or adverse events following an ACS incident. Several studies also performed pathway analysis for finding the biological pathways that contribute to disease pathogenesis. Many of these studies have already shown substantial potential for discovery and understanding. However, as discussed above, attempts to translate these study results into clinical practice have resulted in contradictory results. Considering metabolomics is still early in its scientific evolution, the future is promising with the ongoing technological advances in the field. More importantly, more clinical studies aiming at a system-wide understanding of ACS pathogenesis rather than risk prediction models are the need of the hour.

## Figures and Tables

**Table 1:** A summary of the important advantages and limitations of NMR and MS techniques.

Technique	Advantages	Disadvantages
NMR	Highly reproducible results	Relatively low sensitivity compared to MS
	Provides structural information about the compounds	Only suited for medium to high abundant metabolites (micro-molar range)
	Minimal requirement for sample preparation	Relatively longer data acquisition times compared to MS
	Non-destructive in nature and suitable for multiple analyses of the same sample	Highly pH-sensitive
	Allows investigation of tissue energetics and in vivo metabolism	
	Suitable for compounds which are otherwise difficult to ionize or derivatize	
	Appropriate to use with samples with high salt content, including urine	
	Well-established NMR spectra library to aid data analysis	
	Ability to detect different isomeric products	
GC/MS	Method of choice for the analysis of volatile/non-polar metabolites	Detection of polar metabolites is difficult and needs chemical derivatization.
	Increased signal-to-noise (S/N) ratios and relatively better resolution	Limited metabolome coverage
	Publicly available spectral libraries for compound identification	The high temperature applied in GC/MS can cause degradation or transformation of compounds.
LC/MS	Ability to analyze metabolites with a wide range of polarity, including thermally unstable ones	Not suitable for the analysis of gaseous mixtures
	Quicker and less extensive sample extraction procedures	Decreased sensitivity due to ion suppression
	Suitable for measurement of compounds of lower volatility	Difficulty in distinguishing isomers (both structural and positional) of molecules
	Requires little sample volume	

Abbreviations: NMR, nuclear magnetic resonance spectroscopy; GC/MS, gas chromatography/mass spectrometry; LC/MS, liquid chromatography/mass spectrometry.

**Table 2:** Main findings from the clinical studies of metabolomics in acute coronary syndrome.

No.	First Author, Year	Sample Size	Sampling Time	Specimen/ Technique	Main Findings
1	W Zhong [43], 2021	284 ACS; 130 HC	At the time of hospital admission	Plasma LC/MS	Phenylalanine, arginine, and proline metabolism and synthesis and degradation of ketone bodies are involved in ACS pathogenesis.
2	E Chorell [44], 2021	50 STEMI; 50 NSTEMI; 100 HC	After fasting for 4 h	Plasma GC/MS, LC/MS	Plasma lysophospholipids ratio (LPC:LPE) could predict future risk in STEMI and NSTEMI patients.
3	N Aa [45], 2021	85 MI; 61 non-MI chest pain; 84 HC	Within 6 h of the initial symptom attack	Plasma GC/MS, LC/MS	Patients with MI had elevated plasma levels of deoxyuridine, methionine, and homoserine.
4	H Chen [46], 2021	Discovery: 942 Validation: 493	After fasting for 8 h	Plasma LC/MS	Perturbations in cysteine and methionine metabolism and glycerophospholipid metabolism are associated with CAD severity.
5	A Mehta [47], 2020	Discovery: 454 Validation: 322	After overnight fasting	Plasma LC/MS	Perturbations in tryptophan, lysine, tyrosine, asparagine/aspartate, urea cycle, and the carnitine shuttle metabolism are associated with mortality in CAD patients.
6	J Li [48], 2020	136 NOCAD; 118 AMI	After overnight fasting	Serum LC/MS	23 differential metabolites were identified between AMI and NOCAD, including 12 acylcarnitines, 7 fatty acids, 3 glycerophospholipids, and L-tryptophan.
7	H Jiang [49], 2020	252 ACS	After initial diagnosis of ACS	Serum LC/MS	A total of four metabolites including isoundecylic acid, betaine, 1-heptadecanoyl-sn-glycero-3-phosphocholine, and acetylcarnitine could discriminate stable and vulnerable plaques.
8	A Khan [50], 2020	112 patients at AMI risk; 89 HC	During routine blood collection after overnight fasting	Serum LC/MS	L-homocysteine sulfinic acid, cysteic acid, and carnitine could serve as predictive markers for AMI risk.
9	M Pouralijan Amiri [35], 2020	94 UA; 32 controls (angina, but no CAD)	After coronary angiography	Plasma H-NMR	17 metabolites involved in pathways such as steroid hormone biosynthesis, aminoacyl-tRNA biosynthesis, and lysine degradation could serve as promising biomarkers for UA diagnosis.
10	A Vignoli [32], 2020	825 total, 702 survivors and 123 deceased	24–48 h after the PCI and overnight fasting	Serum H-NMR	Characterization of metabolite–metabolite association, can be used as a potential tool to predict mortality in AMI patients.
11	G Gundogdu [51], 2020	20 STEMI; 15 HC	Within an hour of the initial symptom attack	Serum LC/MS	Malonic acid, maleic acid, fumaric acid, and palmitic acid could be used for the diagnosis of STEMI.
12	A Surendran [52], 2019	27 STEMI	Pre-PCI, 2, 24, and 48 h post-PCI	Plasma LC/MS	Identified lipids and lipid-derived molecules as the major constituents of the altered metabolomic profile prior to PCI and in the follow-up time intervals post-PCI.
13	J Wang [53], 2019	40 UA; 39 HC	Blood samples taken at the same day of inclusion in the study	Plasma LC/MS	27 metabolites, including free fatty acids, amino acids, LPE, LPC, and organic acids, can be used to diagnose UA patients.
14	M Deidda [42], 2019	15 STEMI	Coronary artery blood sampling during PCI	Plasma H-NMR	Coronary blood fingerprint in STEMI patients was represented by choline, phosphocholine, myo-inositol, lysine, ornithine, and 2-phosphoglycerate metabolites.
15	A Vignoli [54], 2019	Training: 80 survivors and 40 deceased Validation: 752 survivors and 106 deceased	24–48 h after the PCI and overnight fasting	Serum H-NMR	Mortality in AMI patients was associated with elevated serum levels of acetone, 3-hydroxybutyrate, mannose, creatinine, acetate, formate, proline, and lower serum levels of valine and histidine.
16	VAM Goulart [55], 2019	15 STEMI; 19 HC	Within 7 h after hospitalization	Plasma LC/MS	STEMI metabolic fingerprint includes perturbations associated with phosphatidylcholines, lysophosphatidylcholines, sphingomyelins, and biogenic amine species.
17	Y Wang [56], 2018	36 ACS; 30 HC	Not specified	Urine LC/MS	Identified fatty acid metabolism, fatty acid β-oxidation, amino acid metabolism, and TCA cycle as critical pathways associated with ACS pathogenesis
18	X Du [57], 2018	96 STEMI with post-PCI AEs; 96 without AEs	Arterial blood before coronary angiography	Plasma LC/MS	Circulating levels of branched-chain amino acids (BCAAs) were associated with the risk of adverse cardiovascular events in STEMI patients.
19	X Du [58], 2018	138 STEMI with AHF; 138 STEMI without AHF	At the time of hospital admission	Plasma LC/MS	Elevated plasma BCAA levels were associated with long-term adverse cardiovascular events in patients with STEMI and AHF.
20	L Huang [59], 2018	44 STEMI (22 LMCAD and 22 non-LMCAD); 22 HC	At the time of hospital admission	Plasma LC/MS	Retinol metabolism was the most perturbed metabolic pathway for the LMCAD phenotype.
21	D Dazhi [41], 2018	45 AMI; 45 chest pain controls (CPCS)	At the time of hospital admission and prior to any medication	Serum H-NMR	Multiple altered metabolic pathways, including the TCA cycle, lipoprotein changes, anaerobic glycolysis, gluconeogenesis, and fatty acid metabolism, characterize AMI patients compared to CPCS.
22	M Kohlhauer [60], 2018	115 STEMI; 26 control patients (SA/NSTEMI)	Immediately after stent deployment	Plasma LC/MS	Increased levels of myocardial succinate are found in STEMI patients.
23	L Zhang [61], 2018	2,324 patients who underwent coronary angiography	Before coronary angiography	Plasma LC/MS	N-acetylneuraminic acid plays a key role during CAD progression.
24	X Yin [62], 2018	20 STEMI; 20 non-ACS patients	Pre-PCI	Plasma LC/MS, ICP/MS	ACS patients are characterized by disturbances in LPC, caffeine, glycolysis, tryptophan, and sphingomyelin metabolism.
25	W Yao [33], 2017	22 UA; 22 HC	Within 24 h after overnight fasting	Serum H-NMR	UA patients are characterized by perturbations in phospholipid and amino acid metabolism.
26	SE Ali [63], 2016	30 STEMI; 15 UA; 15 HC	1–2 h post-chest pain for STEMI patients, before and after angioplasty for UA patients	Serum GC/MS, SPME-GC/MS, H-NMR	Elevated levels of serum hydrogen sulfide could discriminate STEMI patients from UA patients.
27	Y Fan [64], 2016	Discovery: 1086 Validation: 933	Before coronary angiography	Plasma LC/MS	89 differential metabolites were identified between and within different CAD subtypes.
28	X Xu [65], 2015	38 SA; 34 AMI; 71 HC	After overnight fasting	Serum LC/MS	Different lipid classes, including fatty acids, steroids, phospholipids, sphingolipids, and glycerolipids, are associated with CAD progression.
29	L Huang [66], 2016	47 STEMI (23 youth, 24 elderly), 48 healthy controls (24 youth, 24 elderly)	Post-PCI	Plasma LC/MS	The most perturbed metabolic pathway in young STEMI patients was sphingolipid metabolism.
30	K Ameta [34], 2016	65 UA; 62 HC	Within 4 h of onset of angina	Serum H-NMR	Five significantly altered metabolites, namely valine, alanine, glutamine, inosine, and adenine, differentiate UA patients from HC.
31	Z Li [36], 2015	27 UA; 20 HC	In the morning after fasting for 12 h	Urine H-NMR	20 metabolites, including energy metabolism-related metabolites and amino acids, could discriminate UA patients from HC.
32	S Naz [67], 2015	Discovery: 16 STEMI; 16 NSTEMI Validation: 20 STEMI; 28 NSTEMI	Pre-PCI	Serum LC/MS	Carnitine-related compounds and amino acids were differentially present in STEMI and NSTEMI conditions.
33	CM Laborde [68], 2013	Discovery: 35 NSTEACS; 35 HC Validation: 15 NSTEACS; 15 HC	At the onset of the syndrome	Plasma GC/MS, LC/MS	A panel of metabolites consisting of 5-OH-tryptophan, 2-OH-butyric acid, and 3-OH-butyric acid could serve as markers for the early diagnosis of ACS.
34	M Sun [69], 2013	45 UA; 43 atherosclerosis controls	In the morning after overnight fasting	Plasma LC/MS	16 potential endogenous biomarkers for UA were identified including kynurenine.
35	J Teul [70], 2011	19 NSTEACS; 6 HC	Immediately before coronary angiography, day 4, 2 months and 6 months after diagnosis	Plasma GC/MS	27 metabolites including glucose, fructose, myoinositol, pyruvate, lactate, and succinate varied with time following an ACS event.
36	M Vallejo [71], 2009	9 NSTEACS; 10 stable atherosclerosis; 10 HC	In the morning after fasting on the 4th day of hospital stay	Plasma GC/MS	Plasma fingerprinting characterizes a key role for 4-hydroxyproline in ACS.

Abbreviations: STEMI, ST-elevation myocardial infarction; NSTEMI, non-ST-elevation myocardial infarction; NSTEACS, non-ST-elevation ACS; NOCAD, nonobstructive coronary artery disease; AMI, acute myocardial infarction; AHF, acute heart failure; MI, myocardial infarction; SA, stable angina pectoris; UA, unstable angina pectoris; PCI, percutaneous coronary intervention; ACS, acute coronary syndromes; CAD, coronary artery disease; HC, healthy control; LC/MS, liquid chromatography/mass spectrometry; GC/MS, gas chromatography/mass spectrometry; H-NMR, proton nuclear magnetic resonance; SPME, solid-phase microextraction; LPC, lysophosphatidylcholine; LPE, lysophosphatidylethanolamine; TCA, tricarboxylic acid cycle.

**Table 3:** Main findings from the clinical studies of lipidomics in acute coronary syndrome.

No	First Author, Year	Sample Size	Sampling Time	Specimen/ Technique	Main Findings
1	L Zhang [88], 2021	20 STEMI	30 min before PCI; 6, 12, 24, and 72 h after PCI; 1 day before discharge; and 28 days after PCI	Plasma LC/MS	The circulating levels of PGE2, PGD2, and TXA2 were significantly lower at 6 h post-PCI in STEMI patients. The levels of 20-HETE content were significantly higher at 12–72 h post-PCI.
2	J Burrello [89], 2020	7 STEMI; 9 controls	Pre-PCI, and 24 h post-PCI	Isolated EV Plasma LC/MS	The levels of ceramides, dihydroceramides, and sphingomyelins in extracellular vesicles increased in STEMI compared to matched controls and decreased post-PCI.
3	PJ Meikle [90], 2019	47 ACS; 83 stable CAD	Before coronary catheterization	Plasma LC/MS	Venous plasma lipid species was better than traditional risk factors in discriminating ACS from stable CAD.
4	JH Lee [87], 2018	30 CAD, 10 ACS, 10 with stable CAD without ACS	Not specified	Plasma LC/MS	Two LPC species (16:0 and 18:0) were significantly elevated only in the HDL of the ACS group vs. the stable CAD group, whereas PE species (38:5 and 40:5) were elevated in ACS by >2-fold in both HDL and LDL.
5	MJ Gerl [91], 2018	74 ACS, 78 SA, 21 IS, 52 HC	Within the first 24 h of hospital admission	Plasma LC/MS	The levels of LPC and ratios of CE to free cholesterol were decreased in the CVD subjects compared to control subjects.
6	S Anroedh [92], 2018	581 ACS; 155 MACEs	Prior to coronary angiography or PCI	Plasma LC/MS	The circulating ceramides were associated with MACEs independent of clinical risk factors in CAD patients.
7	L Feng [93], 2018	40 STEMI	Pre-PCI, 2 h and 24 h post-PCI	Plasma LC/MS	16 circulating fatty acids were associated with myocardial reperfusion injury.
8	C Garcia [94], 2018	30 ACS; 30 No CAD	Before hospital discharge	Plasma LC/MS	HDL2 subclass from ACS patients was enriched with oxidized polyunsaturated fatty acids.
9	LP de Carvalho [95], 2018	Discovery: 337 Validation: 119	Pre-angiography and within 24 h post-angiography	Tissue, Plasma LC/MS	11 ceramides (C14 to C26) and 1 dihydroceramide (C16) were associated with MACEs in patients with AMI.
10	M Chatterjee [96], 2017	175 symptomatic CAD; 15 HC	During coronary angiography	Platelet LC/MS	Symptomatic CAD patients were characterized by a perturbed platelet lipidome.
11	L Zu [97], 2016	39 MACE; 39 Non-MACE; 39 controls	During coronary angiography	Plasma LC/MS	The plasma level of 19-HETE is useful for the prognosis of ACS after adjustment for clinical risk factors.
12	JM Cheng [98], 2015	162 STEMI; 151 NSTEACS; 261 stable CAD	Prior to coronary angiography	Plasma LC/MS	Plasma ceramide (d18:1/16:0) was associated with vulnerable plaque and 1-year MACE.
13	F Rached [86], 2015	16 STEMI; 10 controls	Within 24 h after diagnosis	Plasma LC/MS	The lipidome of HDL particles were markedly altered in STEMI.
14	I Sutter [85], 2015	23 stable CAD; 22 ACS; 22 HC	Within 12 h of the initial symptom attack	Plasma LC/MS	HDL-plasmalogen levels were inversely associated with both stable and acute CAD.
15	JY Park [99], 2015	140 CAD; 70 HC	After fasting for 12 h	Serum LC/MS	PC containing palmitic acid, DG, SM, and Cer were associated with an increased risk of MI, whereas PE-plasmalogen and PI were associated with a decreased risk.
16	PJ Meikle [100], 2011	60 SA; 80 UA; 80 HC	Not specified	Plasma LC/MS	The study showed that multivariate models using multiple lipid species can stratify unstable and stable CAD patients with improved accuracy compared to traditional risk factors.

Abbreviations: MACE, major adverse cardiac events; STEMI, ST-elevation myocardial infarction; NSTEACS, non-ST-elevation ACS; AMI, acute myocardial infarction; MI, myocardial infarction; SA, stable angina pectoris; UA, unstable angina pectoris; IS, ischemic stroke; PCI, percutaneous coronary intervention; ACS, acute coronary syndromes; CAD, coronary artery disease; HC, healthy control; LC/MS, liquid chromatography/mass spectrometry; LPC, lysophosphatidylcholine; PE, phosphatidylethanolamine; CE, cholesteryl ester; PC, phosphatidylcholine; DG, diacylglycerol; SM, sphingomyelin; Cer, ceramide; PI, phosphatidylinositol; HDL, high-density lipoprotein.

## Abbreviations

ACS: acute coronary syndrome; AMI, acute myocardial infarction; BCAA, branched-chain amino acids; CAD, coronary artery disease; CE/MS, capillary electrophoresis and mass spectrometry; CVD, cardiovascular disease; GC/MS, gas chromatography and mass spectrometry; I/R, ischemia/reperfusion; LC/MS, liquid chromatography and mass spectrometry; LDL, low-density lipoprotein; HDL, high-density lipoprotein; MACE, major adverse cardiac events; MS, mass spectrometry; NMR, nuclear magnetic resonance; NSTEMI, non-ST-segment elevation myocardial infarction; PCI, percutaneous coronary intervention; ROS, reactive oxygen species; STEMI, ST-segment elevation myocardial infarction; UA, unstable angina.
